# Functional gastrointestinal disorders among healthcare professionals—hidden in plain sight?

**DOI:** 10.1002/jgh3.12911

**Published:** 2023-04-27

**Authors:** Gaurav B. Nigam, Jimmy K. Limdi

**Affiliations:** ^1^ Translational Gastroenterology Unit Oxford University Hospitals NHS Trust Oxford UK; ^2^ Section of Inflammatory Bowel Diseases Northern Care Alliance NHS Foundation Trust Manchester UK; ^3^ Manchester Academic Health Sciences University of Manchester Manchester UK

Functional gastrointestinal disorders (FGIDs) are chronic or recurrent gastrointestinal (GI) disorders without a clear cause, often associated with motility disturbance, visceral hypersensitivity, changes in gut microbiota, and immune response.^1^ FGIDs comprising irritable bowel syndrome(IBS), functional dyspepsia(FD), and functional constipation are incompletely understood and with a complex pathophysiology, accounting for up to a third of referrals to gastroenterology clinics, with symptoms such as epigastric pain, early satiety, postprandial fullness, abdominal pain, diarrhea, and constipation.^2^Indeed, nearly half the general population could meet the criteria for a FGID at any given time, what with the considerable overlap among conditions, more than two‐thirds having seen a doctor in preceding 12 months, 40% using regular medication, and up to a third having had potentially unnecessary and avoidable surgery (such as hysterectomy or cholecystectomy), to address their symptoms.^3^ It is now universally acknowledged that FGIDs have multidimensional and often negative effects on patients' personal, psychological, professional, and social well‐being, with ramifications ranging from impaired quality of life to societal costs including (but not limited to) healthcare expenditure and diminished productivity, of similar magnitude to organic gastrointestinal disease, underscoring their fundamental importance to healthcare systems and society at large.^3^, ^4^

The wide prevalence of FGIDs in the general population notwithstanding, less is known about FGIDs among medical and healthcare professionals. Healthcare professionals (HCPs) play a key role in the well‐being of patients, facing a myriad of challenges on a daily basis, ranging from long and irregular working hours, high stress levels, and the need to make timely or critical decisions, often in a high‐pressure environment. Although physicians and nurses do their best to provide outstanding care to their patients, they often do not prioritize their own self‐care, with resultant negative impacts on their physical and mental health.^5^

A web‐based survey from Ontario demonstrated an increased likelihood of IBS (Odds Ratio [OR] 1.32) for every hour of sleep deprivation among resident doctors, after adjustment for age and gender.^**6**^ Similarly, a study from Michigan demonstrated that rotating shift nurses had a significantly higher prevalence of abdominal pain compared with day shift (81% vs. 54%, *P* < 0.0001) and night shift workers (61%, *P* = 0.003), implicating disruption of circadian rhythm from shift work, on‐call shifts, inadequate sleep quality, and psychological stress in the risk of FGID.^7^

Adding further perspective to these observations, in this issue of *JGH Open*, Nagarethinam et al. report a cross‐sectional survey assessing the prevalence and associations of FGIDs among HCPs from a tertiary Australian hospital setting.^8^ Between January 2017 and June 2018, 274 HCPs were surveyed, and data were collected for demographics, medical role, work hours, sleep habits, stress levels (frequency and severity), and dietary factors. Risk factors for FGIDs and their symptoms based on the Rome III criteria were assessed using a self‐report questionnaire (FGID‐Q), and quality of life (QoL) was assessed for its impact on work, leisure, relationships, and social activities.^9^ Responses were scored using Likert scales, numerical scales, and open text questions. Participants were considered to have FGID if they met Rome III criteria. Only FD and IBS were specifically examined in this study.

The study involved 274 respondents (77% female, median age 35 years) consisting mostly of nurses (66%), followed by doctors (17%) and allied health professionals (10%). Of the respondents, 54% had experienced some GI symptoms three or more times per week, with 46% of these reporting concurrent changes in stool frequency and/or consistency. The three most common GI symptoms experienced were early satiety (42%), non‐epigastric/lower abdominal pain (31%), and epigastric pain (22%). FGIDs were identified in 23% of respondents, (2% IBS, 19% FD, and 2% both). Nearly a third (30%) of respondents experienced an adverse impact on their QoL due to GI symptoms.

The investigators identified a number of risk factors associated with GI symptoms and FGIDs. The risk factors for GI symptoms included female sex (58% vs. 38%, [OR] 2.3, *P* = 0.006), Caucasian versus Asian ethnicity (59% vs. 35%, OR 2.7, *P* = 0.002), high stress levels (67% vs. 40%, OR 3.1, *P* < 0.001), and irregular working hours (62% vs. 46%, OR 1.9, *P* = 0.019). Multivariable analysis identified being easily stressed (OR 2.7, *P* = 0.001) and female sex (OR 2.4, *P* = 0.037) as predictors of GI symptoms, while Asian ethnicity was protective (OR 0.42, *P* = 0.027).

For FGIDs, higher prevalence was observed in respondents who often felt stressed (27% vs. 10%, OR 3.4, *P* = 0.013) or felt easily stressed (29% vs. 17%, OR 2.1, *P* = 0.015), as well as in nurses compared with other HCPs (27% vs. 16%, OR 1.9, *P* = 0.049). FGID rates were similar between males (23%) and females (23%). Multivariate modeling showed that being often stressed (OR 4.1, *P* = 0.011), with working as a nurse tending toward significance (OR 2.0, *P* = 0.057), was associated with FGIDs. Intriguingly, GI symptoms and FGIDs were not found to be associated with age, smoking, alcohol intake, sleep deprivation, or dietary factors (including red meat and fruit/vegetable intake).

Nagarethinam and colleagues are to be commended for tackling an under‐researched area of great importance. The incisive identification of factors associated with risk of GI and FGIDs, if not entirely surprising, is sobering. The study findings are concerning as FGIDs can negatively impact HCPs' work and personal life, leading to reduced productivity and burnout.^10^

The high prevalence of FGIDs among HCPs has potentially serious implications for any healthcare system. Firstly, HCPs with FGIDs may experience reduced QOL and work productivity, impacting patient care.^10^, ^11^ For instance, they may need to take time off work or be less focused with patients, with potential risk of medical errors and its undesirable or serious consequences. Often hidden in plain sight is resilience among medical professionals, which underpins the moral fabric of professionalism and commitment.^12^ However, it is entirely plausible that this might delay HCPs from seeking help for assessment of stress or FGIDs and related symptoms. HCPs with FGIDs may consider the perception of stigma and discrimination from colleagues who may not understand the nature and impact of these disorders and in turn act as a deterrent to any referral for assessment.^11^, ^13^ Although considered inversely proportional, the boundaries between resilience and burnout are not immutable.^12^ Compassion satisfaction (the sense of satisfaction, meaning, and joy experienced from caring for people in challenging situations) must consider the delicate balance with fatigue and its consequences as an important determinant for healthcare professionals' QoL and indeed for healthcare itself.^14^ Future studies should examine resilience and burnout as well as factors associated with identification and referral for such symptoms including HCP roles and characteristics of the practice and external environments.

Healthcare systems must address system issues in the clinical care environment, which are needed to reduce burnout and promote physician well‐being. One way to address the issue of FGIDs among HCPs could be to promote healthy lifestyles and stress management. Hospitals and healthcare organizations can encourage regular exercise and offer stress management programs such as mindfulness‐based stress reduction and cognitive‐behavioral therapy.^10^ These interventions can improve the physical and mental health of HCPs, leading to reduced levels of stress, improved sleep quality, and increased work productivity and indeed quality of patient care delivered.

Creating a supportive work environment that promotes open communication and access to mental health services is crucial. HCPs should be encouraged to seek help if they experience symptoms of FGIDs or other health conditions. Mental health programs, such as employee assistance programs, counseling services, and peer support groups, can provide valuable resources for HCPs and improve their well‐being. Additionally, interventions that focus on improving workplace culture, reducing workload, and providing access to counseling and support groups can also contribute to reducing the prevalence of FGIDs among HCPs, ensuring they have the resources needed to effectively perform their roles without experiencing burnout or job dissatisfaction.^15^

This study by Nagarethinam and colleagues and its sobering observations raise several important questions, challenging our perceptions about HCPs, indeed ourselves. The limitations of the study, while reflecting a paucity of literature, make a compelling case for clinicians to pause and consider our perceptions and paradigms regarding what constitutes HCP well‐being and how we address gaps in recognizing and addressing them. We may have only scratched the surface, but as these questions seek better answers, it also fills us with renewed optimism of a better future for those who provide professional expertise and care and for our patients for whom we care.
